# Introduction of cryobiopsies in the diagnostics of interstitial lung diseases – experiences in a referral center

**DOI:** 10.1080/20018525.2016.1274099

**Published:** 2017-01-09

**Authors:** Sissel Kronborg-White, Birgitte Folkersen, Torben Riis Rasmussen, Nina Voldby, Line Bille Madsen, Finn Rasmussen, Venerino Poletti, Elisabeth Bendstrup

**Affiliations:** ^a^ Department of Respiratory Diseases and Allergy, Aarhus University Hospital, Denmark; ^b^ Department of Pathology, Aarhus University Hospital, Denmark; ^c^ Department of Radiology, Aarhus University Hospital, Denmark; ^d^ Department of Diseases of the Thorax, Ospedale Morgagni, Forli, Italy

**Keywords:** Cryobiopsy, diagnostic techniques, respiratory system, idiopathic pulmonary fibrosis, interstitial lung disease

## Abstract

**Introduction**: Transbronchial cryobiopsies (cTBB) has emerged as a new method for obtaining lung tissue biopsies in the diagnosis of interstitial lung diseases (ILDs). Until now, it has been used in a few highly specialized interventional centers and has shown promising results in obtaining a definite diagnosis of ILDs.

**Method**: All patients undergoing a cTBB between November 2015 and June 2016 were included in this case series study. Data on patient demographics, high-resolution computed tomography patterns, size and number of biopsies, histology patterns, the contribution to a confident diagnosis and complications were registered.

**Results**: Thirty-eight patients underwent cTBB in the period. cTBB contributed to the diagnosis in 28 (74%) of the 38 patients. Only few complications were observed; pneumothorax was the most frequent complication (10 patients, 26%). In six patients, local bleeding occurred during the procedure and was easily controlled by a Fogarty catheter balloon and in some cases tranexamic acid.

**Conclusion**: Performing cTBB in the diagnostics of ILDs is a safe and feasible procedure. cTBB resulted in a confident diagnosis in 74% of cases.

## Introduction

Interstitial lung diseases (ILDs) form a heterogeneous group of more than 200 different disorders. The diagnostic process in ILDs is multidisciplinary and typically requires a combination of radiological, pathological and clinical findings to establish a confident diagnosis. Invasive investigations such as bronchoalveolar lavage (BAL), transbronchial biopsies (TBBs) or surgical lung biopsies (SLBs) are often necessary to make a definite diagnosis.[1,2] A firm diagnosis is important for treatment decisions and has in recent years received increasing relevance after the introduction of anti-fibrotic treatment and reports of a detrimental treatment effect of immunosuppressive treatment in subjects with idiopathic pulmonary fibrosis (IPF).[3]. Conventional TBBs with forceps are normally recommended for diffuse perilymphatic or centrilobular diseases such as sarcoidosis, carcinomatous lymphangitis and subacute hypersensitivity pneumonia (HP). The diagnostic yield in more peripheral and heterogeneous disorders such as those having a histological background of usual interstitial pneumonitis (UIP), non-specific interstitial pneumonitis (NSIP) and other complex morphologic features is limited due to sample size and crush artefacts.[4,5] SLB has been the gold standard for many years. However, SLB usually requires general anesthesia and hospitalization and is associated with complications such as bleeding, infection and acute exacerbation. Moreover, the biopsy procedure carries a non-negligible mortality rate, specifically in elderly, fragile patients with reduced pulmonary function or in those where the probability to document a UIP pattern is higher.[6,7] Pneumothorax is part of the procedure and observed in all cases. Therefore, less invasive procedures yielding comparable diagnostic information are warranted.

Transbronchial cryobiopsy (cTBB) has recently emerged as a new method for sampling lung tissue in ILD and it has proven to be associated with promising results.[8–13] The cryosurgical equipment operates by the Joule–Thomson effect, which dictates that a compressed gas released at high flow rapidly expands and creates a very low temperature. The cooling agent – in the vast majority of reports being carbon dioxide – is applied under high pressure through the central canal of the probe. Weight and diameter of cryobiopsies correlate positively with longer freezing time and larger diameters of the cryoprobe.[14] Samples retrieved by this method are significantly larger than by conventional transbronchial forceps and without crush artefacts. Complications like bleeding, pneumothorax and acute exacerbation have been reported, but at a significantly lower rate when compared to SLB.[13,15–18]

We here present the first Danish experiences with cTBB in the diagnosis of ILDs.

## Methods

The use of cTBB in the diagnosis of ILDs at our Center began in November 2015 and was performed by three experienced interventional pulmonologists (BF, TRR, NV) in patients with suspected ILD. One pulmonologist (BF) visited a center with a large experience in cTBB (Ospedale GB Morgagni, Department of Diseases of the Thorax, Forlì, Italy), and on return, the two other pulmonologists were trained to perform the procedure (TRR, NV).

cTBB was carried out during flexible bronchoscopy (Olympus, Tokya, Japan). Cryoprobes (Erbokryo CA, ERBE, Tubingen, Germany) with a diameter of 1.9 and 2.4 mm were used. Bronchoscopy was performed with the patients in general anesthesia. Patients were intubated with an orotracheal tube. Oxygen saturation, blood pressure, ECG and transcutaneous carbon dioxide partial pressure were monitored continuously. Before the cTBB procedure, 0.5–1 g of tranexamic acid adjusted to body weight was administered intravenously to reduce the risk of prolonged bleeding. The bronchopulmonary segment for biopsy was determined prior to the procedure based on a high resolution computed tomography (HRCT) of the chest. BAL for cytological differential count preceded cTBB. A Fogarty balloon, running outside the operating channel of the flexible bronchoscope, was positioned at the entrance of the preselected segmental bronchus. The cryoprobe was introduced into the selected area by fluoroscopic guidance through the channel of the flexible bronchoscope. A distance of approximately 10 mm from the thoracic wall and a perpendicular relation between the thoracic wall and the probe was considered optimal. Once in position, the probe was cooled for five or seven seconds depending on the size of the cryoprobe used and then the flexible bronchoscope and the probe were retracted with the frozen lung tissue attached on the tip of the probe. While the flexible bronchoscope was pulled out, the Fogarty balloon was inflated. The frozen specimen was thawed in isotonic saline and then fixed in formalin. The aim was to take four biopsies. Chest X-ray was performed after the procedure to assess possible pneumothorax.

Exclusion criteria were FVC below 50% of predicted, DLCO below 35% of predicted, body mass index ≥ 35 and cardiac or any other comorbidity that could significantly increase the risk of complications.

HRCT scan were classified according to recent guidelines.[1] In particular, patients with honeycombing changes recognized independently by two radiologists were not enrolled in the study. Radiologists made one hypothesis if they were highly confident with regard to the pattern identified or at maximum two hypotheses (unclassifiable ILD was one allowed option) when the identification of one pattern was not possible. The criteria used in analyzing cryo-samples to recognize the morphological diagnoses were the same as used for SLB. In detail, the UIP pattern was identified with ‘high confidence’ when patchy fibrosis, fibroblastic foci, ± honeycombing were identified. It was reported with ‘low confidence’ when patchy fibrosis without fibroblast foci (± honeycombing) or fibroblastic foci without associated collagenous fibrosis (± honeycombing) were identified.[8,19]

Data on demographics, HRCT patterns, size and number of biopsies, histological patterns, contribution to a confident diagnosis of ILD and complications were registered in a prospective case series study. Data were registered between November 2015 and June 2016.

Data are presented as mean ± standard deviation (SD) or median (range if continuous).

## Results

Thirty-eight patients (16 F/22 M) with ILD underwent bronchoscopy with BAL and cTBB between November 2015 and June 2016. The median age was 61 years (range 29–80). The pulmonary function tests were only slightly reduced. Demographic characteristics are presented in Table 1. HRCT findings are presented in Table 2.

**Table 1.** Patient characteristics at time of inclusion.

Patients demographics	
No. of patients	38
Gender (F/M)	16/22
Age, median, (range)	61 (29–80)
Smoking status (former, current, never, unknown)	21/9/7/1
Pulmonary function	
FEV1 (L)	2.3 ± 0.7
FEV1 % predicted	79% ± 22
FVC (L)	3.2 ± 1.06
FVC % predicted	87% ± 27
DLCO predicted	60% ±15
6MWTD (meters)	477 ± 141

FEV1: forced expiratory volume in 1 s; FVC: forced vital capacity; DLCO: diffusing capacity of the lungs for carbon monoxide; 6MWTD: six-minute walk test distance.

**Table 2.** HRCT findings at time of inclusion.

HRCT hypothesis	No. (%)
NSIP/possible UIP	19 (50)
Subacute HP/DIP	3 (7,9)
EP/NSIP	3 (7,9)
Chronic HP/NSIP	2 (5,3)
Cystic lung disease	2 (5,3)
Alveolar hemorrhage/vasculitis	1 (2,6)
PAP/alveolar hemorrhage	1 (2,6)
PAP/granulomatous lung disease	1 (2,6)
Subacute HP/sarcoidosis	1 (2,6)
NSIP/COP	1 (2,6)
Chronic HP/possible UIP	1 (2,6)
Chronic HP/unclassifiable interstitial fibrosis	1 (2,6)
DIP/RB-ILD	1 (2,6)
NSIP/RB-ILD	1 (2,6)

NSIP: non-specific interstitial pneumonia; UIP: usual interstitial pneumonia; HP: hypersensitivity pneumonia; DIP: desquamative interstitial pneumonia; EP: eosinophilic pneumonia; PAP: pulmonary alveolar proteinosis; COP: cryptogenic organizing pneumonia; RB-ILD: respiratory bronchiolitis related interstitial lung disease.

A median number of four cTBBs were taken (36 patients four biopsies, one patient three biopsies and one patient two biopsies). Thirty-three (87%) of the biopsies were taken from the lower lobe (right side: 31 (94%), left side: 2 (6%)). The mean larger diameter of the biopsies was 6.4 mm ± 2.5 mm. Crush artefacts were identified in only one biopsy. Pleura was present in eight of the 38 patients (21%). Three of the 153 samples contained only bronchial tissue and therefore they were labeled as inadequate.

The histological results are presented in Table 3. The most common pathological pattern identified was UIP (*n* = 10) with high confidence in seven (70%) and low confidence in three (30%) patients, respectively. In four patients with a diagnosis of UIP pattern with high confidence, besides patchy fibrosis and fibroblastic foci, honeycomb changes were identified.

**Table 3.** Histological diagnosis based on cryobiopsies.

Histological diagnosis	No. (%)
UIP	10 (26,3)
– High confidence	7 (70)
– Low confidence	3 (30)
HP	6 (15,8)
– Subacute	3 (50)
– Chronic	3 (50)
Cellular NSIP	5 (13,2)
RB-ILD	3 (7,9)
Fibrotic NSIP	3 (7,9)
Sarcoidosis	2 (5,3)
Lymphoid hyperplasia	1 (2,6)
Nodular bone metaplasia	1 (2,6)
OP/DIP	1 (2,6)
No diagnosis	6 (15,8)

UIP: usual interstitial pneumonia; HP: hypersensitivity pneumonitis; NSIP: non-specific interstitial pneumonia; RB-ILD: respiratory bronchiolitis interstitial lung disease; OP: organizing pneumonia; DIP: desquamative interstitial pneumonia.

After a multi-disciplinary team discussion (MDT) a consensus diagnosis was achieved. The clinical diagnoses can be seen in Table 4.

**Table 4.** Clinical diagnosis after a multidisciplinary team discussion.

Clinical diagnosis	No. (%)
IPF	
– High confidence	8 (21,1)
– Low confidence	2 (5,3)
HP	
– Subacute	3 (7,9)
– Chronic	3 (7,9)
SR-ILD	5 (13,2)
Possible NSIP/possible IPF	2 (5,3)
Idiopathic NSIP	2 (5,3)
Sarcoidosis	2 (5,3)
Drug induced-ILD	2 (5,3)
Vasculitis (ANCA associated)	1 (2,6)
Scleroderma-associated ILD	1 (2,6)
Organizing pneumonia	1 (2,6)
Histiocytosis X	1 (2,6)
LAM	1 (2,6)
Pulmonary nodular lymphoid hyperplasia	1 (2,6)
Dendriform metaplasia	1 (2,6)
Antisynthetase syndrome	1 (2,6)
No diagnosis	1 (2,6)

IPF: idiopathic pulmonary fibrosis; HP: hypersensitivity pneumonitis; SR-ILD: smoking-related interstitial lung disease, including bronchiolitis and RB-ILD; NSIP: non-specific interstitial pneumonitis; ANCA: antineutrophilic antibodies; LAM: lymphangioleiomyomatosis.

The cryobiopsies contributed to a confident diagnosis in 28 of the 38 cases (74%). SLB was recommended in two patients. In the other patients, observation without further invasive investigations was pursued.

**Figure 1.**
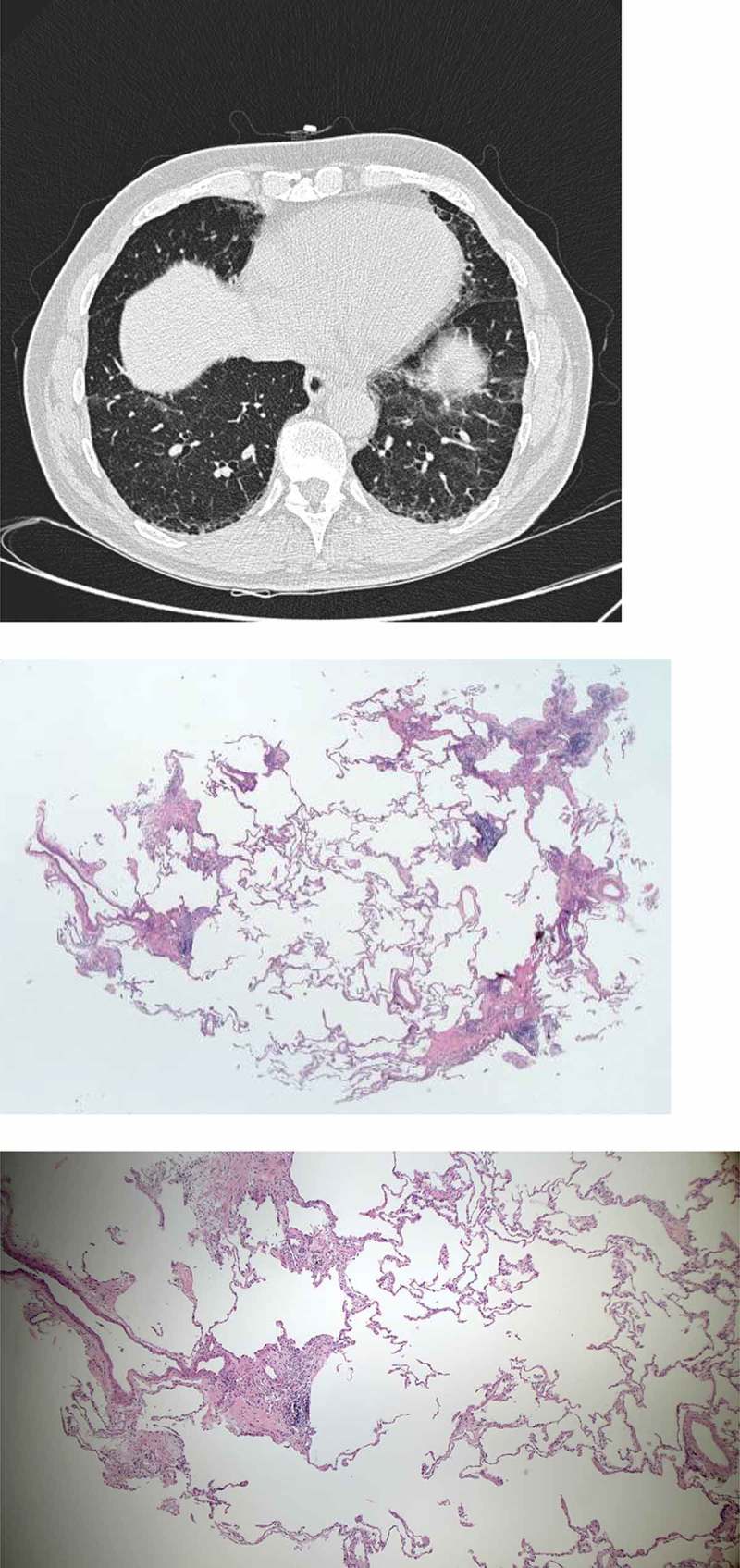
71-year-old male referred for dyspnea. HRCT showing reticulation, ground glass opacity and traction bronchiectasies with basal predominance. Cryobiopsies showing patchy fibrosis, fibroblastic foci and chronic inflammation. The patient was diagnosed with idiopathic pulmonary fibrosis, high confidence.

**Figure 2.**
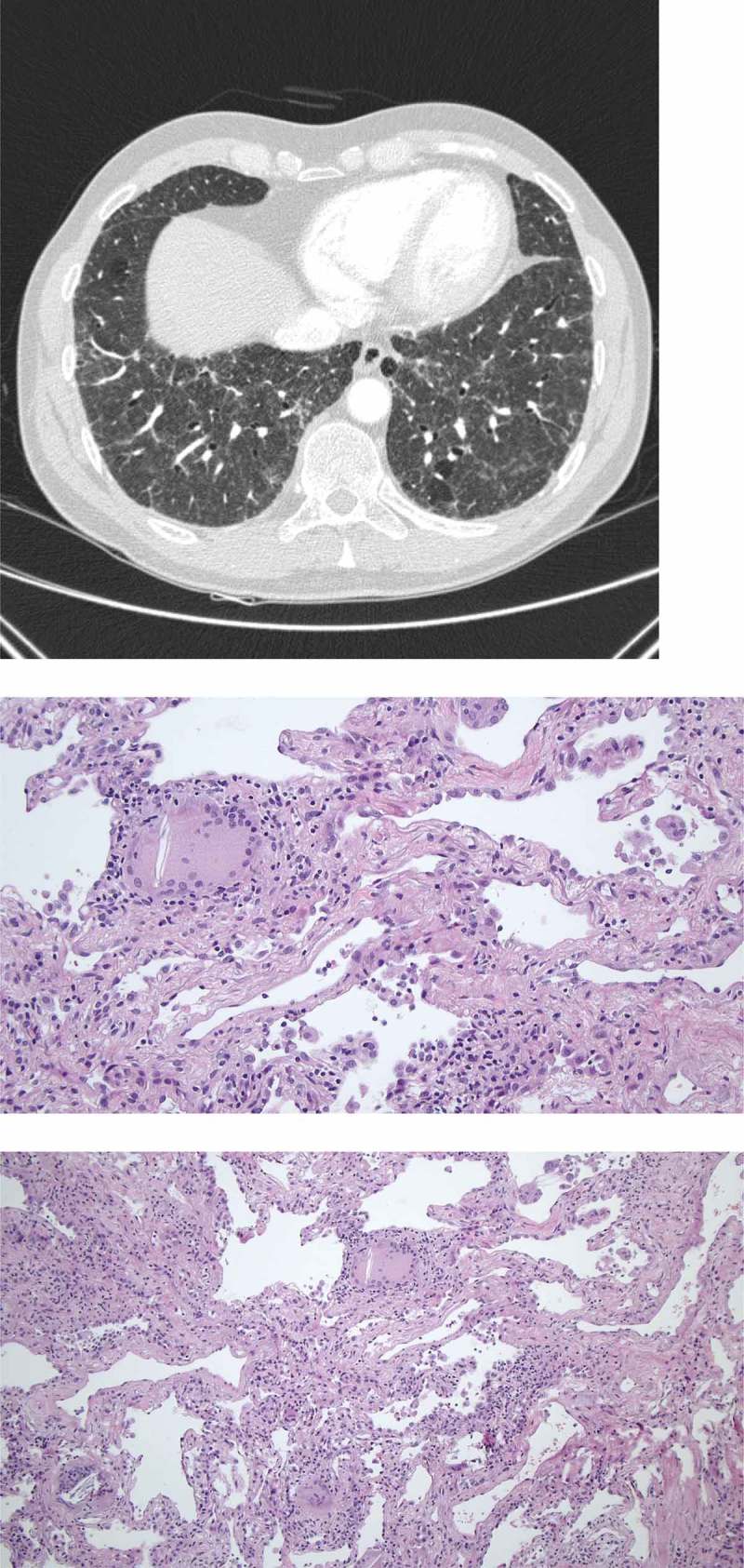
51-year-old male referred for increasing dyspnea and cough. HRCT showing diffuse reticulation, ground glass opacity, traction bronchiectasies and consolidations. Cryobiopsies showing chronic inflammation, fibrosis and granulomas/giant cells. The patient was diagnosed with chronic hypersensitivity pneumonitis.

Of the 38 patients, 20 (53%) presented with a possible UIP pattern on the HRCT. Among these, IPF was the final diagnosis in nine patients, HP in three patients, NSIP in two patients, NSIP/possible IPF in two patients. One had only nodular bone metaplasia, one was diagnosed with drug-induced ILD (venlafaxine), one had a diagnosis of smoking-related interstitial lung disease (SR-ILD) and one patient did not get a diagnosis. cTBB allowed a confident diagnosis to be reached in 80% of the patients with possible UIP pattern on HRCT. Seven patients presented with an HRCT pattern, suggestive for hypersensitivity pneumonitis (for the absence of a cranio-caudal gradient and the presence of reticulation, areas of ground glass opacification and air trapping in at least three lobes). Among these patients, cryobiopsies led to the diagnosis of HP in three patients, two were diagnosed with SR-ILD, one was diagnosed with sarcoidosis and one with IPF. We reached a confident diagnosis in all the patients presenting with HP changes on HRCT. Examples of HRCT scans and histology pictures can be seen in Figures 1 and 2.

In six of the patients, the cryobiopsies showed normal lung tissue or only chronic scanty inflammation. These patients had demographics and HRCT findings highly suggestive of an ILD, but pulmonary function tests were only mildly reduced and HRCT findings were very discrete. These patients were selected for cTBB with the aim of establishing a diagnosis of a mild IPF. In two of the patients we still suspect NSIP/Pos IPF and they are followed with HRCT and pulmonary function tests. SLBs were not considered due to their discrete radiological changes. One patient was submitted to SLB. The samples so obtained showed a UIP pattern and the final diagnosis was IPF. One was diagnosed with SR-ILD after MDT and one was diagnosed with lymphangioleiomyomatosis (LAM) due to the HRCT pattern and elevated vascular endothelial growth factor D. The final diagnosis in one patient was bland chronic alveolar hemorrhage.

None of the patients with NSIP as the histological diagnosis was diagnosed with IPF as the final clinical diagnosis.

Twenty (53%) patients had no complications. One patient (3%) experienced hemoptysis for 10 days after the procedure and was treated with tranexamic acid. In six patients (16%) moderate bleeding occurred during the procedure and it was controlled with the Fogarty balloon, ice water and in two of the patients with tranexamic acid intravenously. Pneumothorax occurred in 10 patients (26%) and in eight patients (21%) a chest tube was inserted. Six out of 10 patients who developed pneumothorax had a diagnosis of UIP pattern on cryo samples. Two patients showed signs of infection after the procedure. One patient was treated with oral antibiotics due to elevated C-reactive protein (CRP) but no fever; the other was treated with intravenous antibiotics due to elevated CRP and fever. No deaths or acute exacerbations of the underlying diseases were observed (Table 5).

**Table 5.** Cryobiopsy complications.

Cryobiopsy complications	No. (%)
Pneumothorax	10 (26)
– Chest tube	2 (5)
+ Chest tube	8 (21)
Bleeding	6 (15)
Infection	2 (5)

## Discussion

We here report the first Danish experiences with cTBB in the diagnosis of ILD. Three pulmonologists experienced in interventional pulmonology performed all procedures and found the technique easy and feasible to implement. A confident diagnosis was obtained in 74% of the patients. The diagnostic yield is lower than that reported in the majority of larger series but this may represent the lack of experience in the new technique;[15] our diagnostic yield is however higher than that recently reported by Ussavarungsi et al. [20] but in that series the smaller cryoprobe was always utilized and the freezing time was between 3 and 5 s, suggesting that the use of larger cryoprobes and longer freezing time may increase the diagnostic yield. The rate of UIP patterns found in this study was 26%, similar to what has been reported in other studies,[9,11] but not as high as reported by Casoni et al. [8]. A UIP pattern was identified with high confidence in eight out of 10 cases, also comparable to other studies.[21] In fact, cryo-samples were large enough to frequently allow the identification of the two histological hallmarks of UIP pattern: patchy fibrosis and fibroblastic foci. Furthermore honeycomb changes were identified in four out of 10 patients with UIP pattern, corroborating the concept that cryo-samples may be sufficient for a sharp identification of this complex histological pattern. The percentage of patients with a UIP pattern included in the study is probably related to the fact that patient selection criteria used in the study were looser: all patients with diffuse parenchymal lung disease on CT scan were included. Cryobiopsies were also very useful in identification of HP when appearing with chronic pattern on the HRCT scan. In our series three cases showed fibrosing inflammation mimicking the UIP pattern, but with the association of scattered granulomas, centrilobular inflammation and fibrosis and scattered areas of organizing pneumonia. Although this study does not compare cryobiopsy results with the information obtained by surgical biopsies, the incidence of chronic hypersensitivity pneumonitis [three out of 38 cases (8%)] is comparable to that described in epidemiologic studies done in Denmark.[22] For patient safety reasons we selected less severely affected patients with correspondingly less affected lung function in the beginning of the study. Also patients with mild ILD where the value of diagnostic certainty is not normally balanced by the risk of performing a surgical lung biopsy were included in this study. After a MDT conference, only two patients were recommended to proceed to SLB. Thus, cTBB avoided the need of surgical lung biopsy in the majority of patients.

Our diagnostic yield was high and resulted in the identification of several specific morphological entities. A confident diagnosis was obtained in 74% of all our patients. Ravaglia et al. [15] achieved a diagnostic yield of 82.8% in 246 patients submitted to cTBB. In SLB, a confident diagnosis was achieved in 98.7% of 148 patients. Thus, even after few procedures, our outcome is similar to what has previously been shown in other ILD centers.

The risks for complications using cTBB were reviewed by Ravaglia et al. [15]. The authors compared the complications associated with cTBB to those observed after SLB, confirming that the mortality due to acute exacerbation is about 0.1% [13,15]. We did not observe any acute exacerbations of the underlying disease in our study. The pneumothorax rate in our study was higher compared to that reported in other series (26%),[13,15] probably reflecting the lack of experience among the pulmonologists in the first period of their training. In fact, if patients were divided into four groups, it is evident that the pneumothorax rate was significantly higher in the first period (four cases of pneumothorax in the first 10 patients) and significantly lower after this first period (two cases of pneumothorax in 10 patients in the other groups). Other factors such as a UIP pattern, cTBB in the left lung and severe fibrotic changes could be considered as predictors of pneumothorax. In the present study, only two patients had cTBB from the left lung and none of those had a pneumothorax. Five of the 10 patients with pneumothorax had a histological UIP pattern on cryobiopsy samples. Our data are in favor of this hypothesis. However, the small number of patients cannot clearly confirm that the histological UIP pattern might be a predictor of increasing pneumothorax rate. Other complications such as moderate bleeding and infection were observed in a minority of patients. Our patients were in general hospitalized for one day for cTBB, compared to at least three days for SLB. Other studies found a hospitalization time for cTBB of 2.6 days and 6.1 days for SLB.[15] Thus, cTBB also seems to be associated with shorter hospitalization time and therefore reduced costs.

## Conclusion

The first Danish experiences with cTBB have been successful and show that cryobiopsies are easy and feasible to perform. The complication rate was low and no serious adverse advents were observed. cTBB can be performed safely in an outpatient setting and results in a confident diagnosis in the majority of patients. cTBB appears to be a safe and less demanding option with a high diagnostic yield compared to SLB.

## Supplementary Material

Supplementary Material

## References

[CIT0001] Raghu G, Collard HR, Egan JJ (2011). An official ATS/ERS/JRS/ALAT statement: idiopathic pulmonary fibrosis: evidence- based guidelines for diagnosis and management. Am J Respir Crit Care Med.

[CIT0002] Poletti V, Ravaglia C, Gurioli C (2016). Invasive diagnostic techniques in idiopathic interstitial pneumonias. Respirology.

[CIT0003] Hilberg O, Simonsen U, du Bois R (2012). Pirfenidone: significant treatment effects in idiopathic pulmonary fibrosis. Clin Respir J.

[CIT0004] Ganganah O, Guo SL, Chiniah M (2016). Efficacy and safety of cryobiopsy versus forceps biopsy for interstitial lung diseases and lung tumors: A systematic review and meta-analysis. Respirology.

[CIT0005] Poletti V, Ravaglia C, Tomassetti S. (2016). Transbronchial cryobiopsy in diffuse parenchymal lung diseases. Curr Opin Pulm Med.

[CIT0006] Hutchinson JP, Fogarty AW, McKeever TM (2016). In-hospital mortality after surgical lung biopsy for interstitial lung disease in the United States. 2000-2011. Am J Respir Crit Care Med.

[CIT0007] Kondoh Y, Taniguchi H, Kitaichi M (2006). Acute exacerbation of interstitial pneumonia following surgical lung biopsy. Respir Med.

[CIT0008] Casoni GL, Tomassetti S, Cavazza A (2014). Transbronchial lung cryobiopsy in the diagnosis of fibrotic interstitial lung diseases. Plos One.

[CIT0009] Pajares V, Puzo C, Castillo D (2014). Diagnostic yield of transbronchial cryobiopsy in interstitial lung disease: a randomized trial. Respirology.

[CIT0010] Hagmeyer L, Theegarten D, Wohlschläger J (2015). The role of transbronchial cryobiopsy and surgical lung biopsy in the diagnostic algorithm of interstitial lung disease. Clin Respir J.

[CIT0011] Kropski JA, Pritchett JM, Mason WR (2013). Bronchoscopic cryobiopsy for the diagnosis of diffuse parenchymal lung disease. Plos One.

[CIT0012] Fruchter O, Fridel L, El Raouf BA (2014). Histological diagnosis of interstitial lung diseases by cryo- transbronchial biopsy. Respirology.

[CIT0013] Colby TV, Tomassetti S, Cavazza A (2016). Transbronchial cryobiopsy in diffuse lung disease: update for the pathologist. Arch Pathol Lab Med.

[CIT0014] Poletti V, Casoni GL, Gurioli C (2014). Lung Cryobiopsies: a paradigm shift in diagnostic bronchoscopy?. Respirology.

[CIT0015] Ravaglia C, Bonifazi M, Wells AU (2016). Safety and diagnostic yield of transbronchial lung cryobiopsy in diffuse parenchymal lung diseases: A comparative study versus video- assisted thoracoscopic lung biopsy and a systematic review of the literature. Respiration.

[CIT0016] Dhooria S, Sehgal IS, Aggarwal AN (2016). Diagnostic yield and safety of cryoprobe transbronchial lung biopsy in diffuse parenchymal lung diseases: systematic review and meta-analysis. Respir Care.

[CIT0017] Hagmeyer L, Theegarten D, Treml M (2016). Validation of transbronchial cryobiopsy in interstitial lung disease - interim analysis of a prospective trial and critical review of the literature. Sarcoidosis Vasc Diffuse Lung Dis.

[CIT0018] Tomassetti S, Wells AU, Costabel U (2016). Bronchoscopic lung cryobiopsy increases diagnostic confidence in the multidisciplinary diagnosis of idiopathic pulmonary fibrosis. Am J Respir Crit Care Med.

[CIT0019] Colby TV, Carrington CB, Thurlbeck WM, Churg AM (1995). Interstitial lung disease. Pathology of the lung.

[CIT0020] Ussavarungsi K, Kern RM, Roden AC (2016). Transbronchial cryobiopsy in diffuse parenchymal lung disease: retrospective analysis of 74 cases. Chest.

[CIT0021] Tomassetti S, Cavazza A, Colby TV (2012). Transbronchial biopsy is useful in predicting UIP pattern. Respir Res.

[CIT0022] Hyldgaard C, Hilberg O, Muller A (2014). A cohort study of interstitial lung diseases in central Denmark. Respir Med.

