# Supplementary material for: Introduction of cryobiopsies in the diagnostics of interstitial lung diseases – experiences in a referral center
Source: Eur Clin Respir J. 2017 Jan 9;4(1):1274099. doi: 10.1080/20018525.2016.1274099 (PMC5328381; doi:10.1080/20018525.2016.1274099)
Supplement: Supplementary Material [file zecr_a_1274099_sm2226.doc]

**Supplementary**

| Patient no. | Radiological first /second hypothesis | Histological diagnosis | MDT diagnosis | Contribution of cryobiopsies to diagnosis |
| --- | --- | --- | --- | --- |
| 1 | DIP/RB-ILD | Cellular NSIP | SR-ILD | Yes |
| 2 | cHP/Possible UIP | cHP | cHP | Yes |
| 3 | EP/NSIP | Nodular lymphoid hyperplasia | Idiopathic nodular hyperplasia | Yes |
| 4 | NSIP/Possible UIP | UIP, high confidence | IPF, high confidence | Yes |
| 5 | NSIP/Possible UIP | UIP, high confidence | IPF, high confidence | Yes |
| 6 | cHP/NSIP | UIP, low confidence | IPF, low confidence | Yes |
| 7 | NSIP/Possible UIP | Normal | NSIP/Possible IPF | No |
| 8 | NSIP/Possible UIP | Dendriform metaplasia | Dendriform metaplasia | Yes |
| 9 | NSIP/Possible UIP | Fibrotic NSIP | NSIP | Yes |
| 10 | NSIP/COP | Cellular NSIP | Drug-induced ILD | Yes |
| 11 | NSIP/Possible UIP | UIP, low confidence | IPF, low confidence | Yes |
| 12 | NSIP/Possible UIP | Normal, bronchiolitis | SR-ILD | Yes |
| 13 | NSIP/Possible UIP | Subacute HP | Subacute HP | Yes |
| 14 | NSIP/Possible UIP | Normal | NSIP/Possible IPF | No |
| 15 | cHP/unclassifiable interstitial fibrosis | cHP | cHP | Yes |
| 16 | NSIP/Possible UIP | Cellular NSIP | Drug-Induced ILD  (venlafaxine) | Yes |
| 17 | NSIP/Possible UIP | Cellular NSIP | NSIP | Yes |
| 18 | NSIP/cHP | cHP | cHP | Yes |
| 19 | NSIP/Possible UIP | Subacute HP | Subacute HP | Yes |
| 20 | Alveolar hemorrhage/Vasculitis | Chronic interstitial inflammation NOS | ANCA-vasculitis | No |
| 21 | PAP/alveolar hemorrhage | Fibrotic NSIP | SR-ILD | No |
| 22 | Cystic lung disease (homogeneous cysts- LAM?) | Normal | LAM | No |
| 23 | NSIP/Possible UIP | UIP, high confidence | IPF, high confidence | Yes |
| 24 | Subacute HP/DIP | RB-ILD | SR-ILD | Yes |
| 25 | NSIP/Possible UIP | Normal | No diagnosis | No |
| 26 | Cystic lung disease (irregular changes- Histiocytosis X?) | Cellular NSIP | Histiocystosis X | No |
| 27 | EP/NSIP | OP/DIP | OP | No |
| 28 | Subacute HP/DIP | Subacute HP | Subacute HP | Yes |
| 29 | Subacute HP/DIP | RB-ILD | SR-ILD | Yes |
| 30 | NSIP/Possible UIP | UIP, low confidence | IPF, low confidence | Yes |
| 31 | PAP/granulomaous lung disease | Sarcoidosis | Sarcoidosis | Yes |
| 32 | NSIP/RB-ILD | RB-ILD | Ssc-ILD | No |
| 33 | NSIP/Possible UIP | UIP, high confidence | IPF, high confidence | Yes |
| 34 | NSIP/Possible UIP | UIP, high confidence | IPF, high confidence | Yes |
| 35 | EP/NSIP | fibrotic NSIP | Antisynthetase syndrome | No |
| 36 | NSIP/Possible UIP | UIP, high confidence | IPF, high confidence | Yes |
| 37 | Subacute HP/sarcoidosis | Sarcoidosis | Sarcoidosis | Yes |
| 38 | NSIP/Possible UIP | UIP, high confidence | IPF, high confidence | Yes |

MDT: multidisciplinary team, DIP: desquamative interstitial pneumonia, RB-ILD: respiratory bronchiolitis interstitial lung disease, NSIP: non-specific interstitial pneumonitis, SR-ILD: smoking-related interstitial lung disease, cHP: chronic hypersensitivity pneumonitis, UIP: usual interstitial pneumonia, EP: eosinophilic pneumonia, IPF: idiopathic pulmonary fibrosis, , COP: cryptogenic organising pneumonia, HP: hypersensitivity pneumonitis, NOS: not otherwise specified, ANCA: antineutrophilic antibodies , LAM: lymphangioleiomyomatosis, PAP: pulmonary alveolar proteinosis, OP: organising pneumonia, Ssc-ILD: scleroderma-associated interstitial lung disease,
